# The Gastric Microbiota Invade the Lamina Propria in *Helicobacter pylori*‐Associated Gastritis and Precancer

**DOI:** 10.1111/hel.70016

**Published:** 2025-02-26

**Authors:** Harriet J. Giddings, Ana Teodósio, Jordanne Jones, Jack L. McMurray, Kelly Hunter, Riad Alame, Isaac Gardiner, Zainab Abdawn, William Butterworth, Ian R. Henderson, Jeffrey A. Cole, Claire D. Shannon‐Lowe, Amanda E. Rossiter‐Pearson

**Affiliations:** ^1^ Institute of Microbiology and Infection University of Birmingham Birmingham UK; ^2^ Department of Microbes, Infection and Microbiomes, School of Infection, Inflammation and Immunology, College of Medicine and Health University of Birmingham Birmingham UK; ^3^ Birmingham Tissue Analytics, University of Birmingham Birmingham UK; ^4^ Rheumatology Research Group University of Birmingham Birmingham UK; ^5^ Queen Elizabeth Hospital University Hospital Birmingham NHS Foundation Trust Birmingham UK; ^6^ Department of Immunology and Immunotherapy, School of Infection, Inflammation and Immunology, College of Medicine and Health University of Birmingham Birmingham UK; ^7^ Institute for Molecular Biosciences University of Queensland Brisbane Australia

**Keywords:** gastric adenocarcinoma, *Helicobacter pylori*, microbiome, spatial biology

## Abstract

**Background:**

Stomach cancer is the fourth leading cause of cancer‐related deaths worldwide. *Helicobacter pylori* is the main risk factor for gastric adenocarcinoma (GAC), yet the precise mechanism underpinning this association remains controversial. Gastric intestinal metaplasia (GIM) represents the precancerous stage and follows *H. pylori‐*associated chronic gastritis (CG). Sequencing studies have revealed fewer *H. pylori* and more non‐ *H. pylori* bacteria in GAC. However, the spatial organization of the gastric microbiota in health and disease is unknown.

**Materials and Methods:**

Here, we have combined RNA in situ hybridization and immunohistochemistry to detect *H. pylori,* non‐ *H. pylori* bacteria, and host cell markers (E‐cadherin, Mucins 5AC and 2) on tissue sections from patients with CG (*n* = 15) and GIM (*n* = 17).

**Results:**

Quantitative analysis of whole slide scans revealed significant correlations of *H. pylori* and other bacteria in CG and GIM. In contrast to sequencing studies, significantly fewer non‐ *H. pylori* bacteria were detected in *H. pylori‐*negative patients. Importantly, whilst *H. pylori* exclusively colonized the gastric glands, non‐ *H. pylori* bacteria invaded the lamina propria in 6/9 CG and 8/10 GIM *H. pylori* ‐positive patients. A rapid and cost‐effective modified Gram stain was used to confirm these findings and enabled detection of non‐ *H. pylori* bacteria in GIM samples.

**Conclusions:**

The invasion of the gastric lamina propria by non‐ *H. pylori* bacteria during *H. pylori‐*associated CG and GIM represents an overlooked phenomenon in cancer progression. Further work must determine the mechanisms underlying the synergistic roles of *H. pylori* and other bacteria in carcinogenesis. This observation should redirect attempts to prevent, diagnose, and treat GAC.

## 1 Introduction

Gastric adenocarcinoma (GAC) is the fourth leading cause of cancer‐related deaths, accounting for 7.7% of cancer mortalities worldwide [1]. *Helicobacter pylori* infection is associated with approximately 70% of GAC cases and the global prevalence of *H. pylori* infection is 43.9% [2]. Correa's cascade describes the histopathological changes to the gastric mucosa during progression to GAC [3]. *H. pylori* first triggers chronic gastritis (CG), which can lead to gastric intestinal metaplasia (GIM), dysplasia and finally GAC. Although genotypes of *H. pylori* that encode the virulence factors CagA, VacA and HtrA [4] are more associated with severe disease outcomes, the mechanism by which a minority of *H. pylori* infections (1%) develop GAC is not fully understood. In recent years, multiple 16S ribosomal RNA (16S rRNA) and metagenomic sequencing studies have profiled the human gastric microbiota during health and *H. pylori‐*associated disease [5, 6, 7, 8]. The proposed model is that the healthy human stomach harbors a distinct microbial community structure and that upon *H. pylori* infection, this community structure shifts towards a dominance of *H. pylori* in CG. As carcinogenesis progresses, multiple other bacterial species displace *H. pylori* and dominate the GAC microbiota. However, sequencing studies are often prone to issues with contamination [9] and do not offer spatial resolution. A longstanding question is whether the gastric microbiota play a causative or correlative role in GAC. Here, we provide high‐resolution spatial images of *H. pylori* and non‐ *H. pylori* bacteria in patients with *H. pylori* ‐positive or negative CG and GIM. Importantly, we provide direct evidence of non‐ *H. pylori* bacteria invading the lamina propria in the early to middle stages of Correa's cascade, suggesting that non‐ *H. pylori* bacteria might play a synergistic role in the cause of these disease states.

## 2 Materials and Methods

### 2.1 Sample Preparation and Pretreatment Protocol for Automated Tissue Staining

Punch biopsy gastric corpus tissue samples were collected from consenting patients at the Queen Elizabeth Hospital, Birmingham, fixed in formalin and prepared at the Human Biomaterial Resource Centre (HBRC), University of Birmingham by embedding in paraffin (Ethics #17–285) and retrieved from archived stocks at HBRC. Exclusion criteria were previous *H. pylori* eradication therapy, antibiotic treatment 4 weeks prior to endoscopy, tissue from the cardia, fundus, or antrum and patients under 30 years old. Routine histologic evaluation of hematoxylin and eosin (H&E)‐stained gastric mucosal sections was used for *H. pylori* diagnosis. Tissue sections (4 μM thick) were prepared for RNAscope and immunohistochemistry (IHC) staining, using a Leica BOND RX Fully Automated Research Stainer. Prior to staining, sections were prepared in the BOND RX, following three short protocols: (1) Deparaffinization, rehydration, hydrogen peroxide and distilled water wash, (2) Heating to 100°C in target retrieval buffer ER2 (pH 9; AR9640) for 45 min and manual washing with distilled water followed by 100% ethanol and drying at 60°C and (3) Incubation in protease III solution for 30 min, followed by a final wash in distilled water.

### 2.2 Optimization of RNAscope Treatments and Immunohistochemistry Antibody Concentrations for 5‐Plex Automated Staining

Automated RNAscope C1 and C2 probes against *H. pylori* (ACD‐Bio, #542938) and Eubacteria (ACD‐Bio, #464468), respectively, were first tested on healthy colon tissue in a fluorescent multiplex assay, using an RNAscope LS Multiplex Fluorescent Reagent Kit (ACD Bio, #322800), following the standard protocol recommended for this platform. Pretreatment of tissue slides for automated tissue staining with lysozyme was not used as this was found to affect tissue integrity. However, automated tissue staining with lysozyme (micro bacteria detection protocol provided by ACD‐Bio) and a 3‐plex panel (E‐Cadherin and RNAscope probes against *H. pylori* and Eubacteria) was used on all samples to ensure the bacterial signal was not underestimated in comparison to 5‐plex stained images (data not shown).

A positive and negative control probe section was used on every RNAscope run to validate and assess its quality and the sensitivity of the assay. The bacterial gene *dapB* (ACD Bio, #312038) was used as a negative control to confirm the absence of background noise, and a cocktail of housekeeping genes *polr2A* C1, *ppiB* C2 and *ubc* C3 (ACD Bio, #320868) was used as a positive control to validate the detection of the signal and the tissue integrity. *H. pylori* gene sequences can bind to both 16S rRNA probes against *H. pylori* and Eubacteria, whilst non‐ *H. pylori* bacteria stain with only the Eubacteria probe.

All antibodies used in IHC steps were optimized prior, using chromogenic DAB staining of healthy colon tissue in addition to a Leica Bond Polymer Refine Detection kit (Leica, #DS9800). Antigen retrieval was tested using pH 6 (Leica Bond TM Epitope Retrieval 1, #AR9961) and pH 9 (Leica Bond TM Epitope Retrieval 2, #AR9640) buffers by heating to 100°C for 20 min. Three different dilutions were tested for each antibody, as recommended by the manufacturer. Ideal staining pattern and intensity was assessed and approved by a pathologist, whereby slides were then used as reference throughout the validation process. All antibodies were then tested for compliance with the RNAscope pretreatment, to ensure stability after protease III digestion. Once each antibody was assigned an Opal fluorophore, single fluorescence assays were directly compared against DAB‐stained colon tissue to optimize Opal concentration. To assess epitope stability during the following heat steps and to define the order of the addition of antibodies in the multiplex sequence, each antibody was tested individually in the different positions of the panel. Further probe and antibody information can be found in Table S2.

### 2.3 Automated 5‐Plex Co‐RNAscope In Situ Hybridization/Immunohistochemistry Protocol

The automated RNAscope Multiplex Fluorescent LS assay (ACD Bio, #322800) was conducted using a Leica BOND RX according to manufacturer's instructions, incubating the sections with C1 and C2 probes against *H. pylori* and Eubacteria, respectively, for 2 h. An automated IHC staining protocol was immediately followed to fluorescently label E‐cadherin, MUC5AC and MUC2. Between each staining cycle, a heat‐induced stripping step with pH 6 solution was added. Images were acquired using a Vectra Polaris^TM^ multispectral whole slide scanner. Exposure times on the Vectra Polaris Slide scanner for the DAPI, 480, 520, 570, 620, and 690 channels were 1.13 ms, 2.47 ms, 36.06 ms, 5.61 ms, 29.58 ms, and 7.46 ms, respectively.

### 2.4 Counterstaining and Section Visualization

Sections were counterstained with spectral DAPI (Akoya Biosciences) and mounted with ProLong Diamond Antifade Mountant, according to the manufacturer's instructions. Mounted sections were stored in the dark at 4°C until viewing. During optimization steps, sections were visualized using a Zeiss LSM 900 confocal microscope or Mantra 2 Quantitative Pathology Digital Workstation (Akoya Biosciences) and final images were obtained on a Vectra Polaris^TM^ multispectral whole slide scanner (Akoya Biosciences) and saved as a .qptiff file. Images were viewed at 40× magnification using Phenochart Whole Slide Viewer with PhenoImager HT (Akoya Biosciences) or open source QuPath software (Version 0.4.3) [10]. All .qptiff image files were spectrally unmixed by importing and stamping in Phenochart Whole Slide Viewer (Akoya Biosciences), unmixed and exported in InForm software (Akoya Biosciences) and finally restitched as a BIGTIFF file using Visiopharm software (Visiopharm, Hørsholm, Denmark).

### 2.5 Qualitative and Quantitative Image Analysis

For quantitation of target markers in whole slide tissue sections, manual image analysis was conducted using QuPath. For each whole slide scan, tissue regions were first annotated and defined as a region of interest (ROI). Tissue detection was then performed based on the average values of all channels using pixel thresholders. A pixel thresholder was used to calculate mean tissue area (μM^2^), which was exported. Separate pixel thresholders were then created for each individual Opal channel, corresponding to *H. pylori* , Eubacteria, MUC5AC or MUC2. The thresholders were saved, and average area annotation measurements (μM^2^) were obtained for each channel. To account for *H. pylori* double staining and detection in both the *H. pylori* and Eubacteria channels, Eubacteria area was quantified by calculating total Eubacteria channel area minus combined *H. pylori* and Eubacteria channel areas. For each whole slide scan, average percentage area coverage of each marker of interest was calculated by dividing individual channel area over total tissue area x 100. Data were exported from Excel to GraphPad Prism 9 (Version 9.5.1). For qualitative scoring of bacterial invasion, images were viewed using the Phenochart Whole Slide Viewer (Akoya Biosciences) or QuPath. Invasion was scored, whereby 0 = no invasion, 1 = sparse invasion, 2 = moderate invasion (patches of bacteria across sample) and 3 = high invasion (multiple clear regions of bacterial invasion across sample). A Mann–Whitney test was used for statistical analysis in which * indicates *p* < 0.05, ** indicates *p* < 0.01, *** indicates *p* < 0.001 and ns indicates nonsignificant. Data are presented as median ± SEM for each group.

### 2.6 H&E and Modified Gram Staining

Additional sections were stained with H&E for further inflammation analysis and scoring by an independent pathologist. A modified Gram stain was also followed for staining of bacteria in additional tissue sections [11].

## 3 Results

### 3.1 Localization of *H. pylori* and the Gastric Microbiota in Chronic Gastritis and Gastric Intestinal Metaplasia

**FIGURE 1:**
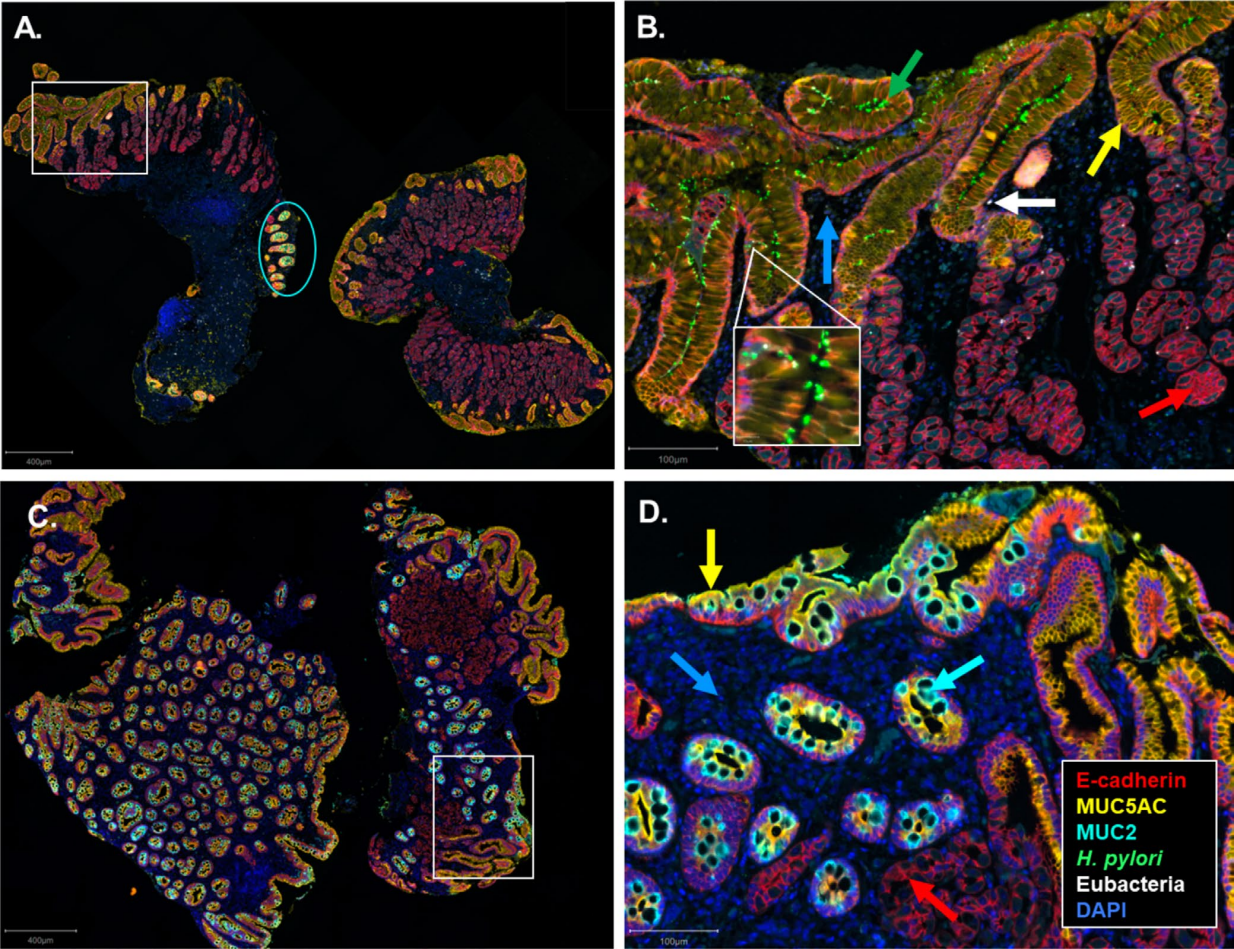
Spatial localization of *H. pylori* and non‐ *H. pylori* bacteria in GIM revealed by RNAscope in situ hybridization (ISH) and immunohistochemistry (IHC). Whole slide scans of stained patient tissue sections were obtained using a Vectra whole slide scanner. Images were spectrally unmixed, viewed and quantified using QuPath. (A & C) A representative whole slide scan of a GIM *H. pylori‐*positive (A) or *H. pylori‐*negative (C) section showing RNAscope ISH probes “ *H. pylori* ” and “Eubacteria” to detect *H. pylori* (green) and non‐ *H. pylori* bacteria (white), respectively. IHC staining against E‐cadherin (red), MUC5AC (yellow) and MUC2 (turquoise) are also shown. (B, D) A higher magnification of regions highlighted with a white box from panels A and C, respectively. The colored arrows show the indicated markers and a region of MUC2 is highlighted within the cyan circle in Panel A.

**FIGURE 2:**
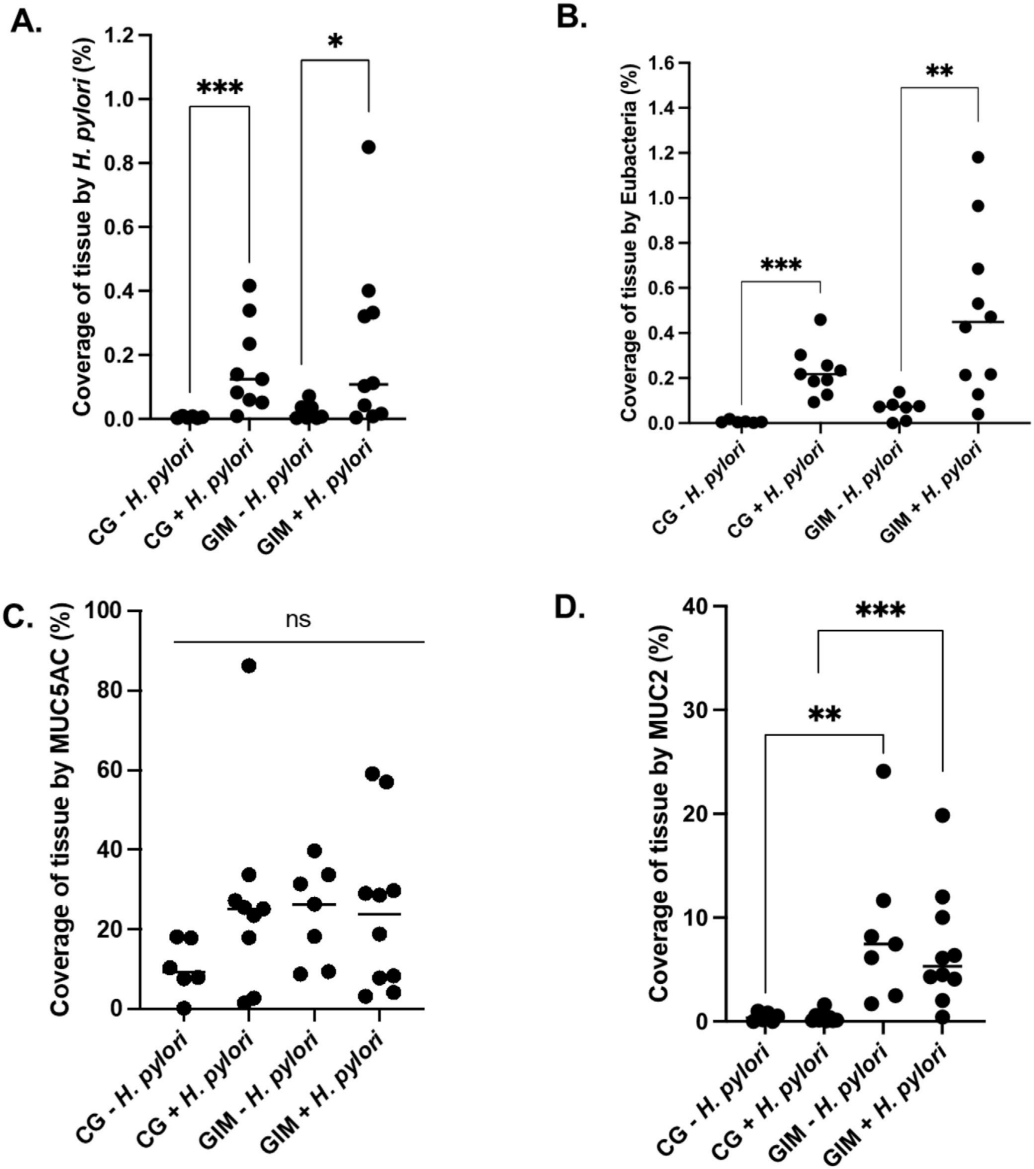
Quantification of target markers in tissue from whole slide scans, using QuPath. The percentage coverage of tissue for each patient group is shown for *H. pylori* (A), Eubacteria (B) MUC5AC (C) and MUC2 (D). Horizontal bars indicate the median for each indicated patient group. A Mann‐Whitney test was used to determine significance where * = *p* < 0.05, ** = *p* < 0.01, *** = *p* < 0.001 and ns = nonsignificant.

Due to anatomical differences and unique microenvironments within the gastric mucosa driving microbial variation [12], archived formalin‐fixed paraffin‐embedded (FFPE) tissue blocks from only gastric corpus tissue were retrieved from the HBRC (*n* = 32) for the purpose of this study. The associated clinical pathology reports confirmed samples were *H. pylori‐*positive CG (*n* = 9) or GIM (*n* = 10) and *H. pylori‐*negative CG (*n* = 6) or GIM (*n* = 7). H&E‐stained tissue sections were prepared from the archived FFPE gastric corpus tissue blocks. Again, using routine histologic evaluation, an independent pathologist confirmed *H. pylori* status and graded all samples for inflammation (Table S1). There was no significant difference in inflammation between *H. pylori‐*positive and *H. pylori‐*negative samples (data not shown). To detect *H. pylori* or non‐ *H. pylori* bacteria, sections were stained using RNAscope in situ hybridization (ISH) probes against *H. pylori‐*specific or conserved sequences of the 16S rRNA gene, respectively. The probe “Eubacteria” was used to detect non‐ *H. pylori* bacteria. IHC enabled detection of the host cell markers E‐cadherin, Mucins 5AC (MUC5AC) and 2 (MUC2) using automated tissue staining for CG and GIM samples. Goblet cells, present only in GIM, secrete MUC2, whereas MUC5AC is found in both healthy and diseased gastric mucus. Representative images of GIM and CG samples are presented in Figure 1 and Figure S1, respectively. Quantification of indicated markers are shown in Figure 2. Comparable levels of MUC5AC were observed across the patient groups and, as expected, there was a significant increase in MUC2 in GIM patients (Figure 2 C&D). Consistent with previous studies [13, 14], *H. pylori* exclusively colonized the gastric glands (Figure 1B). The presence of Eubacteria correlated with *H. pylori* infection in CG and GIM but were significantly reduced in the absence of *H. pylori* infection (*n* = 13) (Figure 2B). This contrasts with the current dogma that there is a unique microbiota in *H. pylori‐*negative individuals [5]. Interestingly, there was an increase in levels of Eubacteria in *H. pylori‐*positive GIM samples compared with *H. pylori‐*positive CG. Although this was not statistically significant, this raised the possibility that the presence of GIM‐specific MUC2 could provide intestinal‐like microniches within the stomach that promote bacterial colonization. As such, we observed colocalization of non‐ *H. pylori* bacteria with MUC2 in 2/10 *H. pylori‐*positive and 1/7 *H. pylori‐*negative GIM patients (Figure S2).

### 3.2 Association of *H. pylori* With Invasion of Non‐ *H. pylori* Bacteria to the Lamina Propria

**FIGURE 3:**
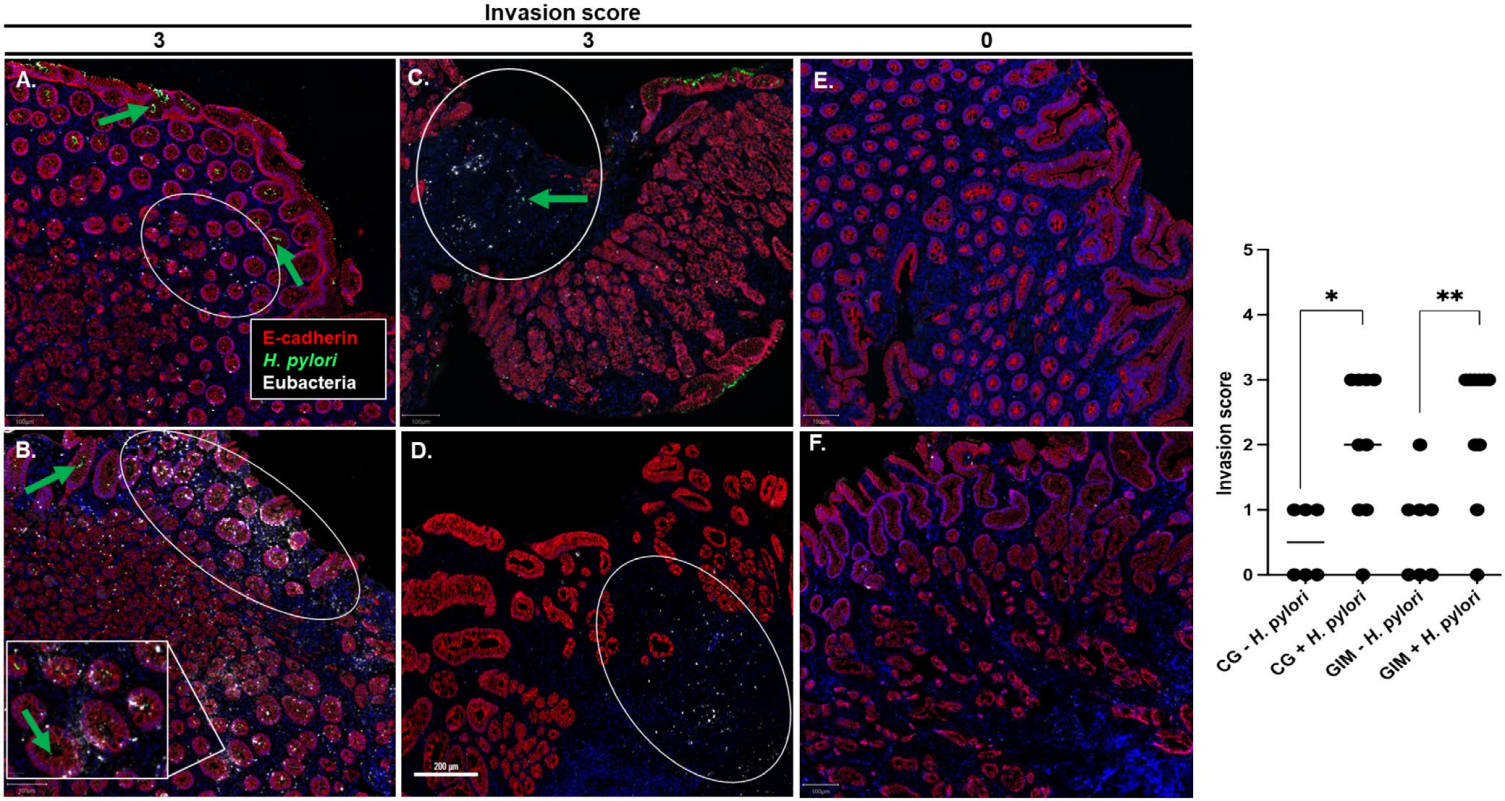
Non‐ *H. pylori* bacteria invade the gastric lamina propria in CG and GIM. Whole slide scans were obtained using a Vectra whole slide scanner. Images were spectrally unmixed and viewed using QuPath. (A–F) Representative images showing Eubacterial (white) invasion in patients with *H. pylori*‐positive CG (A–B), GIM (C–D) or *H. pylori* ‐negative CG (E) and GIM (F). Only E‐cadherin (red), *H. pylori* (green), Eubacteria (white) and DAPI (blue) are shown for visualization purposes. Arrows indicate the target markers. Eubacterial invasion is highlighted in white ovals for CG and GIM. A rare occurrence of *H. pylori* invasion within the lamina propria is indicated by the green arrow in (C). Qualitative invasion ISH scoring of Eubacterial invasion was determined for each patient as follows; 0 = no invasion, 1 = sparse, 2 = moderate and 3 = high invasion. Invasion scores for representative images are indicated above each column and qualitative scores for each patient group are shown in (G). Horizontal bars indicate the mean for each indicated patient group. A Mann–Whitney test was used to determine significance where * = *p* < 0.05 and ** = *p* < 0.01.

Bacterial invasion is associated with multiple diseases of the gastrointestinal tract, causing damage to the epithelial architecture by triggering inflammatory responses [15, 16, 17]. Whilst *H. pylori* colonized the gastric glands, non‐ *H. pylori* bacteria invaded the lamina propria in 6/9 patients with *H. pylori‐*associated CG and 8/10 *H. pylori‐*positive patients with GIM (Figure 3A–D). Qualitative scoring of Eubacterial invasion showed significantly more invasion in *H. pylori‐*positive CG and GIM than *H. pylori‐*negative CG and GIM (Figure 3G). Representative images of *H. pylori‐*positive CG and GIM patients with an invasion score of 3 are shown in Figure 3A–D *H. pylori‐*negative patients with an invasion score of 0 can be seen in Figure 3E&F. Very few Eubacteria are seen in samples with an invasion score of 0. It is most likely that these rare occurrences of Eubacteria in the stomach represent transient bacteria, rather than stable colonization [18]. Invasion of *H. pylori* into the lamina propria is very rare across all patient samples, yet an instance of this can be seen in Figure 3C. Representative images of samples with invasion scores of 1–2 can be seen in Figure S3.

### 3.3 Visualization of Non‐ ***H. pylori*** Bacteria Using a Modified Gram Stain

**FIGURE 4:**
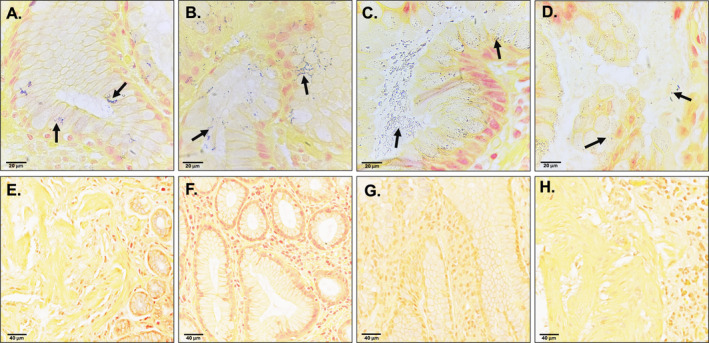
A modified Gram stain detects non‐ *H. pylori* bacteria in GIM. Archived FFPE biopsy tissue from *H. pylori‐*positive GIM patients (*n* = 4) were obtained from the Human Biomaterials Resource Centre (HBRC). After applying a modified Gram stain, slides were viewed at 100× magnification with oil immersion using a light microscope. Images A‐D show a single representative image for each GIM *H. pylori* ‐positive patient. Images E‐H show a region on the same patient where bacteria are not present. Black arrows in Panels A‐D indicate purple stained bacteria.

Next, we aimed to confirm bacterial invasion at the cellular level in patients with *H. pylori‐*positive CG (*n* = 4) or GIM (*n* = 4). As can be seen in Figure 4A–D, microniches of Gram‐positive bacteria could be identified in all 4 *H. pylori‐*positive GIM patients. Visualization of Gram‐negative bacteria with this technique was not clear and H&E‐stained sections were superior for identifying *H. pylori* (data not shown). Bacteria could not readily be identified in *H. pylori‐*positive CG patients using this technique (data not shown). This is consistent with the lower levels of non‐ *H. pylori* bacteria that were detected in CG patients in comparison to GIM patients (Figure 2B). Although further work is required to understand the biological consequences of invasive bacteria in carcinogenesis, this modified Gram stain technique could be used alongside the current histological diagnostic tools to stratify patients at higher risk for developing GAC.

## 4 Discussion

Sequencing technologies have enabled characterization of the human microbiota in health and disease in multiple organs. However, applying 16S rRNA PCR‐based sequencing to low biomass samples, such as the skin and the stomach, can introduce sampling and technical errors in comparison to higher biomass samples [9] and do not offer spatial resolution. Here, we have circumvented these issues by applying advanced imaging technologies to directly visualize the gastric microbiota in CG and GIM. By combining detection of targeted sequences within the bacterial 16S rRNA gene with immunostaining against host cell markers E‐cadherin, MUC5AC, and MUC2 we show that *H. pylori* exclusively occupies the gastric glandular niche, which is in agreement with previous studies [13, 14]. We have also shown the presence of significantly more non‐ *H. pylori* bacteria in *H. pylori‐*infected CG and GIM patients, whereas there are barely detectable levels in *H. pylori* ‐negative CG patients and only slightly more present in *H. pylori‐*negative GIM patients. This is in contrast to sequencing studies, which suggest a distinct microbiome exists in *H. pylori‐*negative disease states [5, 6, 7, 8]. These discrepancies may be due to many factors contributing to an over‐representation of the gastric microbiota using sequencing techniques, such as amplification‐based methods capturing bacterial DNA remnants, contamination issues [9] and detection of transient rather than persistent bacteria [18]. Indeed, this study highlights the crucial need for researchers to use a combination of sequencing [19], spatial profiling, and culture techniques to fully resolve the ecology of the gastric microbiota during health and disease. The most important observation made in this study was that non‐ *H. pylori* bacteria invaded the lamina propria in 67% of *H. pylori‐*positive CG and 80% of *H. pylori‐*positive GIM. The high prevalence of invasive bacteria in *H. pylori‐*positive patients amongst this cohort of patients was surprising. However, this could be explained by these patients being symptomatic and therefore attending for further clinical investigations. Our preliminary studies using intestinal‐type GAC tissue from gastrectomy samples highlighted that comparisons between CG and GIM gastric punch biopsy samples and gastrectomy samples are incredibly difficult to make with spatial biology approaches. The size, stage and molecular subtypes of GAC samples demands that three‐dimensional reconstructions of consecutive tissue sections are required to fully resolve intratumoral microbial communities. As such, these samples were excluded from this study until this comprehensive analysis of GAC samples can be made.

The gastrointestinal tract provides an important barrier to pathogen invasion. Translocation of bacteria and their antigens/metabolites across the intestinal epithelial barrier is associated with gastrointestinal infections and a range of diseases, such as inflammatory bowel diseases and metabolic diseases [15, 16, 17]. Activation of immune cells in the lamina propria drives pathology in these disorders via the production of pro‐inflammatory cytokines and reactive oxygen species. Thus, it is conceivable that invasive bacteria within the gastric lamina propria synergistically activate the immune response in *H. pylori‐*associated CG and GIM. Further work is underway to triangulate the immune response to invasive bacteria within the gastric mucosa.

Given that these archived gastric tissue samples were retrieved via the local tissue bank (HBRC), we did not have access to the associated *H. pylori* clinical isolates from these patients. Recently, Sharafutdinov and colleagues reported that *H. pylori* strains encoding the trimeric form of the HtrA serine protease, which proteolytically cleaves the cell junction proteins occludin, claudin‐8 and E‐cadherin, are associated with a higher GAC risk than strains encoding the monomeric form [4]. It is therefore conceivable that individuals infected with strains of *H. pylori* that secrete the trimeric HtrA cause the gastric epithelial cell barrier to become “leakier”, thereby facilitating invasion of bacteria to the lamina propria. However, it is also possible that *H. pylori‐*associated inflammation can cause disruption to the epithelial barrier by direct and indirect interactions between gastric epithelial cells and mucosal immune cells [20]. Thus, we propose that *H. pylori* facilitates opportunistic invasion of the lamina propria by transiting microbes. Further work is required to compare bacterial invasion in patients that are infected with strains of *H. pylori* encoding the trimeric or monomeric HtrA. Unfortunately, we did not have access to healthy patients for endoscopy and so the microbial landscape in the healthy, *H. pylori‐*negative gastric mucosa is yet to be determined using imaging‐based approaches. Additionally, the prevalence and precise identity of invasive bacteria during the carcinogenic cascade must be determined in a larger cohort of patients.

Interestingly, we have also shown that the levels of non‐ *H. pylori* bacteria increased from CG to GIM (Figure 2B) and that microniches of MUC2 and non‐ *H. pylori* bacteria colocalization are apparent in GIM tissue samples (Figure S2). The region of non‐ *H. pylori* bacteria shown in Figure S2A represents the largest single region of non‐ *H. pylori* bacteria detected amongst all patient samples. Although the levels of Eubacteria was relatively low in this patient (0.14%), it is conceivable that this microniche of bacterial colonization could be due to their use of the proton pump inhibitor, Rabeprazole (Table S1). However, our relatively low number of patients with known PPI use (Table S1) limits our ability to correlate the use of PPI with changes in the gastric microbiota. Nonetheless, our data provides the first observation of non‐ *H. pylori* bacteria with MUC2 within the gastric mucosa. Further work is required to understand whether these unique precancer microenvironments contribute to the estimated 1%–10% of GIM patients who progress to developing GAC [21]. Limitations of this study were that 94% of patients were male, cases of autoimmune gastritis within the *H. pylori* ‐negative CG samples is unknown and PPI use is only known for a small number of patients within this cohort (Table S1). Further studies should address these important considerations, particularly the use of proton pump inhibitors, when assessing the role of *H. pylori* and the microbiota in gastric cancer.

Clinically, GIM has been termed the “point of no return”, given that antibiotic eradication of *H. pylori* in GIM provides only minimal benefit to a patient's risk of developing GAC in comparison to treatment of *H. pylori‐*associated CG [21]. Additionally, patients with GIM are monitored for progression to GAC with 3‐yearly endoscopic surveillance due to a lack of convincing evidence on the use of biomarkers [21]. Here, we show that a rapid and cost‐effective modified Gram stain could identify non‐ *H. pylori* bacteria in GIM, whilst it was less superior at visualizing *H. pylori* than H&E staining (data not shown). Therefore, further work could focus on optimizing the modified Gram stain to better visualize Gram‐negative bacteria, or, modified Gram staining in conjunction with H&E staining of consecutive tissue sections could be utilized for the diagnosis of invasive bacteria and *H. pylori*, respectively. Although future studies must determine whether invasive bacteria drive progression to GAC, it is possible that this represents a novel biomarker for GAC and that antibiotic treatment of GIM patients to eradicate both *H. pylori* and invasive bacteria could be implemented as a preventative treatment for GAC.

In conclusion, we have observed that invasive bacteria are associated with *H. pylori* infection in the early and middle stages of Correa's carcinogenic cascade and therefore represent an attractive target for microbiome‐based interventions in the prevention, diagnosis, and management of GAC.

## Author Contributions

Conceptualization: C.D.S.‐L. and A.E.R.‐P. Methodology: H.J.G, A.T., J.J., J.L.M, K.H., R.A., I.G., W.B. and A.E.R.‐P. Data analysis: H.J.G., A.T., J.J., J.L.M., K.H., Z.A., I.R.H., J.A.C. and A.E.R.‐P. Writing: H.J.G., J.A.C. and A.E.R.‐P. Funding acquisition: A.E.R.‐P.

## Conflicts of Interest

The authors declare no conflicts of interest.

## Supporting information


Figure S1.

Figure S2.

Figure S3.

Tables S1–S2.

## Data Availability

The data that support the findings of this study are available from the corresponding author upon reasonable request.
