# Supplementary material for: The Gastric Microbiota Invade the Lamina Propria in Helicobacter pylori‐Associated Gastritis and Precancer
Source: Helicobacter. 2025 Feb 26;30(1):e70016. doi: 10.1111/hel.70016 (PMC11865006; doi:10.1111/hel.70016)
Supplement: Supplementary file 1 — Figure S1. [file HEL-30-e70016-s004.pdf]

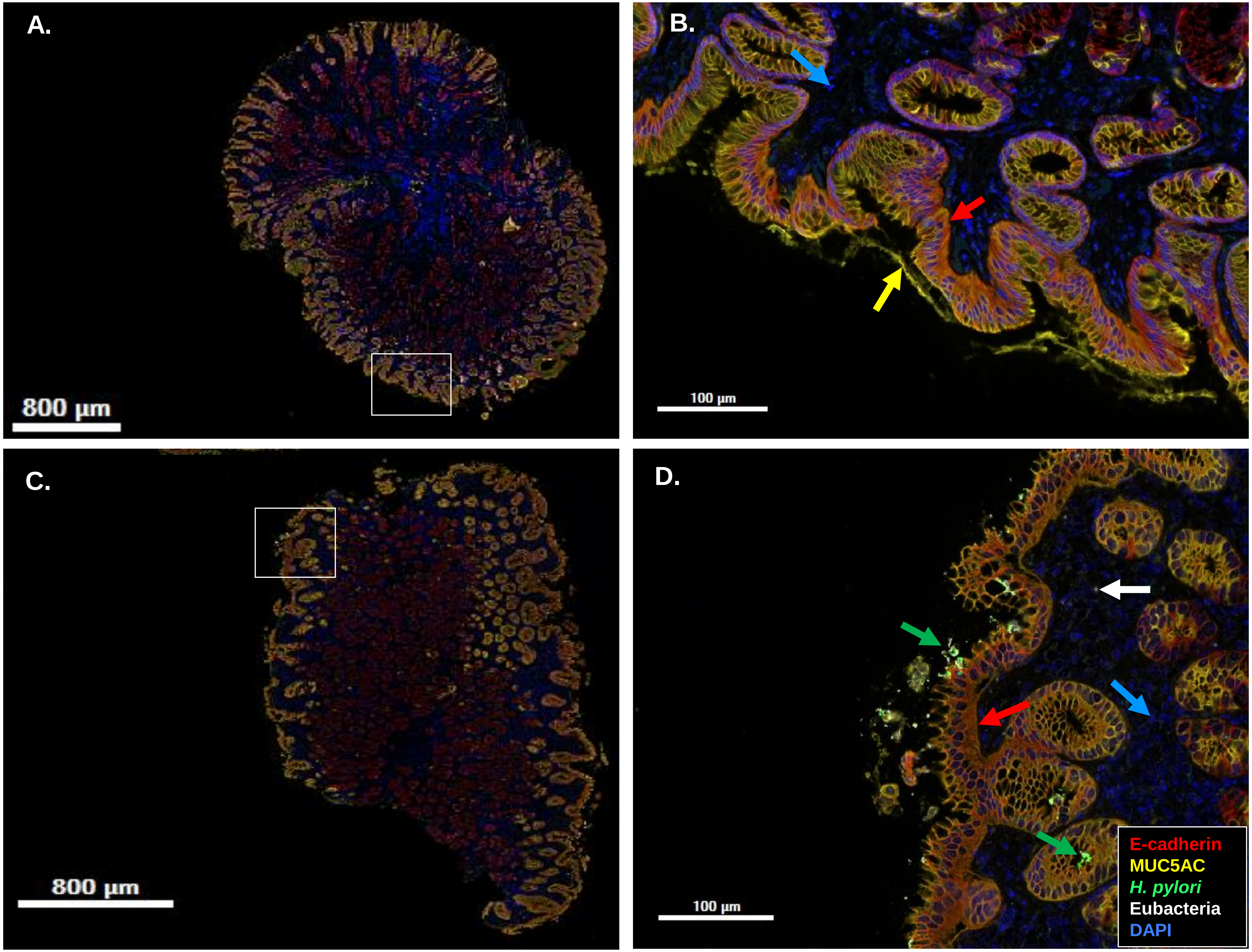

Figure S1. Spatial localisation of *H. pylori* and non-*H. pylori* bacteria in CG revealed by RNAscope *in situ* hybridisation (ISH) and immunohistochemistry (IHC). Whole slide scans of stained patient tissue sections were obtained using a Vectra whole slide scanner. Images were spectrally unmixed, viewed and quantified using QuPath. **A & C)** A representative whole slide scan of a CG *H. pylori*-negative (A) or *H. pylori-positive* (C) section showing RNAscope ISH probes '*H. pylori'* and 'Eubacteria' to detect *H. pylori* (green) and non-*H. pylori* bacteria (white), respectively. IHC staining against E-cadherin (red) and MUC5AC (yellow) are also shown. **B & D)** A higher magnification of regions highlighted with a white box from panels A and C, respectively. The coloured arrows show the indicated markers.
