# Supplementary material for: The Gastric Microbiota Invade the Lamina Propria in Helicobacter pylori‐Associated Gastritis and Precancer
Source: Helicobacter. 2025 Feb 26;30(1):e70016. doi: 10.1111/hel.70016 (PMC11865006; doi:10.1111/hel.70016)
Supplement: Supplementary file 2 — Figure S2. [file HEL-30-e70016-s002.pdf]

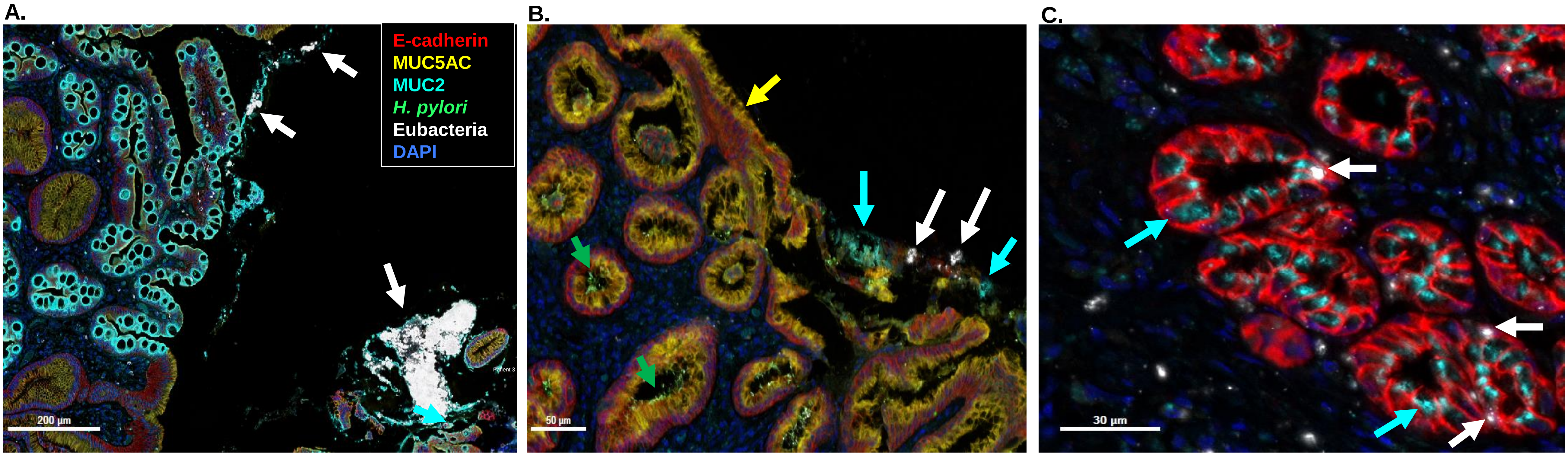

Figure S2. Co-localisation of non-*H. pylori* bacteria and Muc2 in GIM. (A-C) Whole slide scans of stained patient tissue sections were obtained using a Vectra whole slide scanner. Images were spectrally unmixed, viewed and quantified using QuPath. *H. pylori-negative* **(A)** or *H. pylori*-positive **(B-C)** GIM patient samples are shown. RNAscope *in situ* hybridisation probes '*H. pylori'* and 'Eubacteria' were used to detect *H. pylori* (green) and non-*H. pylori* bacteria (white), respectively. Immunohistochemistry staining against E-cadherin (red), MUC5AC (yellow) and MUC2 (turquoise) are also shown. The coloured arrows show the indicated markers.
