# Supplementary material for: The Gastric Microbiota Invade the Lamina Propria in Helicobacter pylori‐Associated Gastritis and Precancer
Source: Helicobacter. 2025 Feb 26;30(1):e70016. doi: 10.1111/hel.70016 (PMC11865006; doi:10.1111/hel.70016)
Supplement: Supplementary file 3 — Figure S3. [file HEL-30-e70016-s001.pdf]

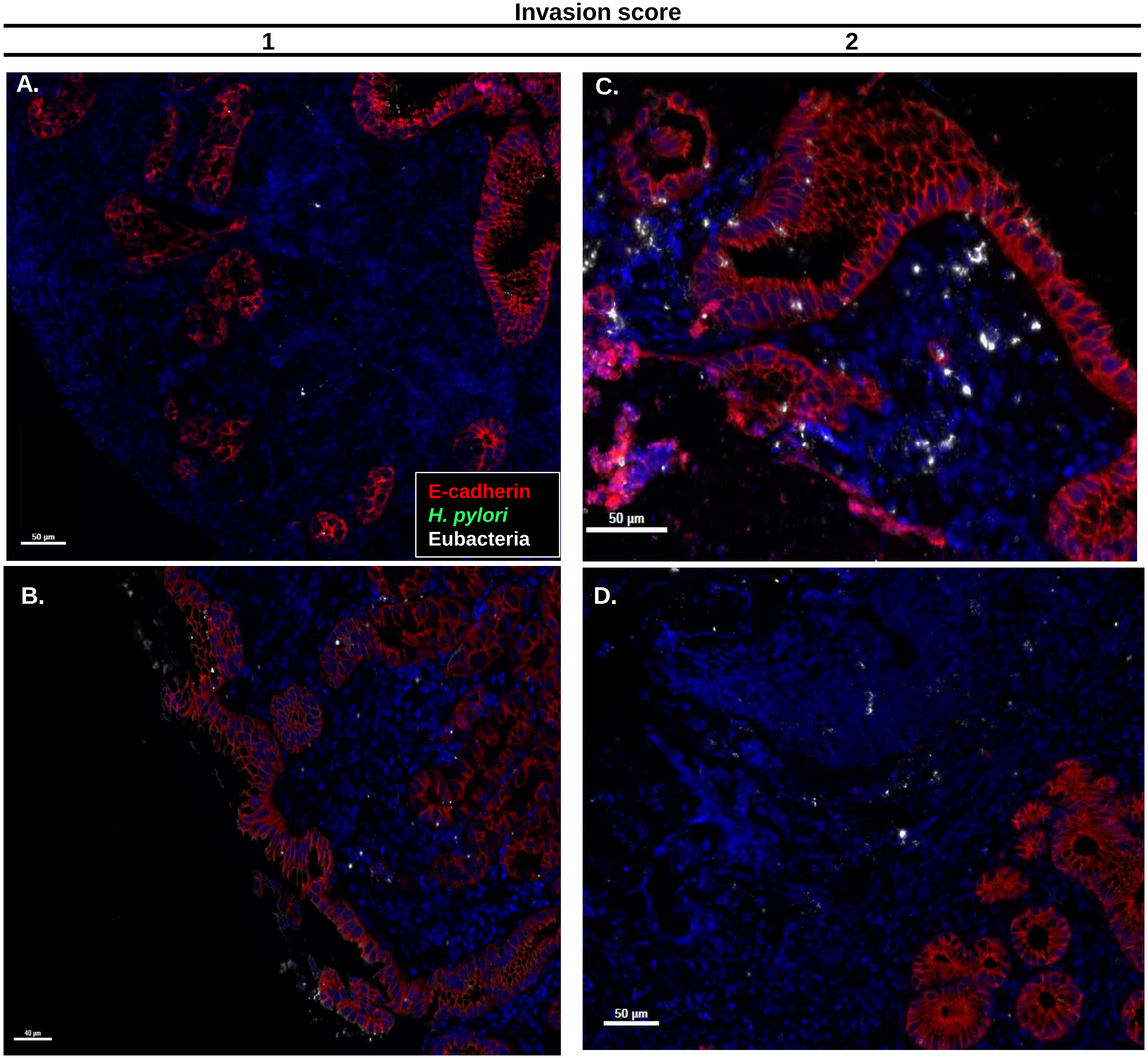

Figure S3. Representative images of scoring system for invasion of non-*H. pylori* bacteria to the gastric lamina propria. Whole slide scans were obtained using a Vectra whole slide scanner. Images were spectrally unmixed and viewed using QuPath. **A-D)** Representative images showing Eubacterial (white) invasion in patients with *H.* pylori-positive CG (A-B) or GIM (C-D). Eubacterial invasion was determined for each patient as follows; 1 = sparse and 2 = moderate invasion. Invasion scores for each image is indicated at the top of each column. Only E-cadherin (red), *H. pylori* (green), Eubacteria (white) and DAPI (blue) are shown for visualisation purposes.
