# Supplementary material for: The Gastric Microbiota Invade the Lamina Propria in Helicobacter pylori‐Associated Gastritis and Precancer
Source: Helicobacter. 2025 Feb 26;30(1):e70016. doi: 10.1111/hel.70016 (PMC11865006; doi:10.1111/hel.70016)
Supplement: Supplementary file 4 — Tables S1–S2. [file HEL-30-e70016-s003.docx]

**Supplementary Table 1. Patient demographics, clinical inflammatory grade and Eubacterial invasion scores**

CG; chronic gastritis, GIM; gastric intestinal metaplasia. PPI; Proton pump inhibitor.

**Supplementary Table 2. Automated 5-plex RNAScope probe and IHC antibody information**

| **Marker** | **Marker type** | **Supplier** | **Clone** | **Cat. No.** | **Marker dilution** | **Fluorophore** | **Fluorophore cat. No.** | **Fluorophore dilution** |
| --- | --- | --- | --- | --- | --- | --- | --- | --- |
| *H. pylori* | RNAScope C1 | ACD | N/A | N/A | RTU | 520 | FP1487001KT | 1:150 |
| Eubacteria | RNAScope C2 | ACD | N/A | N/A | 1:50 | 620 | FP1495001KT | 1:150 |
| E-cadherin | Antibody | Cell Signalling Technology | 24E10 | 3195S | 1:250 | 690 | FP1497001KT | 1:200 |
| MUC2 | Antibody | Novus Biologicals | Polyclonal | NBP1-31231 | 1:1000 | 480 | FP1500001KT | 1:150 |
| MUC5AC | Antibody | Invitrogen | 2-25LE | MA119346 | 1:1000 | 570 | FP1488001KT | 1:150 |
